# Microstructure and Mechanical Properties of an Ultrasonic Spot-Welded Aluminum-to-Aluminum Joint: Response to Interlayer Thickness

**DOI:** 10.3390/ma12030369

**Published:** 2019-01-24

**Authors:** Zeng-Lei Ni, Fu-Xing Ye

**Affiliations:** 1 School of Materials Science and Engineering, North China University of Water Resources and Electric Power, Zhengzhou 450045, China; 2 School of Materials Science & Engineering, Tianjin University, Tianjin 300072, China; fxyepaper@163.com

**Keywords:** aluminum, ultrasonic spot welding, particle interlayer thickness, mechanical property

## Abstract

To enhance the mechanical strength of an ultrasonic spot-welded Al/Al joint, an Al 2219 particle interlayer was placed between the two Al sheets during the ultrasonic spot welding process. The effects of the interlayer thickness on the microstructure and mechanical performances of the joints were systematically investigated. The results showed that, the optimum thickness of the Al 2219 particle interlayer was 10 μm, which was beneficial to enhance the weld interface temperature up to 402 °C. The bonding interface of Al/Al 2219 with a wave-like shape was sound, and no significant diffusion layer occurred. The peak lap shear tensile strength (~84.8 MPa) was obtained, which was 36% higher than that (~67.3 MPa) for the joint without the Al 2219 particle interlayer. The strengthening mechanism is caused by the increase of plastic deformation and contact areas in the weld interface.

## 1. Introduction

Al/Al joints have been widely used in the fields of power device module packing, automobile electronic technologies, and solar power controlling. Bakavos et al. [1] reported that Al alloys possess high conductivity, resulting in challenges when joining similar or dissimilar materials. Haddadi [2] confirmed that the conventional fusion welding process easily led to weld defects, such as hot cracking, high levels of welding deformation, and poor weldability. However, a laser welding technique has been successfully applied to joining Al alloys [3,4]. Barnes et al. [5] showed that bonding and riveting increases the costs of surface preparation and adds consumption products, respectively. Peng et al. [6] confirmed that resistance spot welding makes the application of aluminum alloys difficult because of the low strength, high conductivity, and tendency to degrade of the electrodes. One particular challenge has been the high consumption of energy of the resistance spot-welded aluminum alloy joints (~50–100 kJ per weld), as reported by Jahn et al. [7]. Bakavos et al. [1] demonstrated that friction stir spot welding has the advantage of low energy consumption (~3–6 kJ per weld), but the weld time is remarkably long (~2–5 s). Ultrasonic spot welding (USW) as a further spot welding technique has been paid more attention. Chen et al. [8] confirmed that USW is a solid phase joining technique and that under the combination effects of high shear frequency vibration and clamping force, two metal sheets can be joined by such a technique. Daniels [9] noted that USW was first introduced for thin foils joining, wire bonding, and tube sealing in the 1950 s. It was used to join thicker metal sheets (such as Al, Cu, NiTi, Fe, and Ni) due to advancements in the welding system technique [10,11,12,13]. In comparison with friction stir spot welding and resistance spot welding, USW has low energy consumption (~0.6–1.5 kJ per weld), because the major heat generation is at the weld interface, not on the top surface of the specimen [14]. In addition, Bakavos et al. [15] showed that USW also possesses a shorter weld cycle (typically < 0.5 s). Therefore, USW is a promising spot welding process for fabricating Al/Al joints.

A few studies about aluminum alloy joining have been conducted. Lu et al. [16] investigated the relative motion of aluminum alloy sheets, sonotrode tip, and anvil while employing an in situ velocity measurement technique. The results showed that in the stick stage, when the metal sheets and sonotrode tip vibrate at the same velocity, is crucial to obtaining sound joints, as well as the fracture mode. Jahn et al. [7] reported that the effects of anvil cap geometry on the lap shear failure load and weld microstructures are only slight. Mirza et al. [17] showed that a distinct necklace type structure with fine equiaxial grain was generated at the weld interface, which is attributed to mechanical interlock and the appearance of dynamic recrystallization. Baboi et al. [18] showed that the problem of the sticking phenomenon between the horn and the top sheet could be solved by using a copper buffer sheet. Ji et al. [19] showed that the deformation theories were dynamic recovery and dynamic recrystallization. Shin et al. [20] reported that the phenomenon of weld zone thinning is produced when the welding energy input is high, which has significantly harmful affects on the mechanical strength and fracture mode of the joints. However, there have been no new solutions proposed regarding this trouble, and no studies about regulating the microstructure of the weld interface and enhancing the mechanical strength of the USWed Al/Al joints by changing the frictional slip behavior in the weld interface have been reported in the existing literature.

In the previous studies, it was reported that the appropriate thickness of the Cu foil interlayer could improve the mechanical properties of USWed joints by reducing the amount of brittle intermetallic compounds [21,22]. Wang et al. [23] stated that hard metal particles could enhance the friction coefficient under plane contact. Elangovan et al. [24] confirmed that by increasing the friction coefficient of the weld interface, the temperature of the weld interface increased. Moreover, the weld interface temperature was a key factor of the weld formation because of the active effects of the right weld interface temperature on the improvement of the weldability. Based on the above, in this study, an economical and effective method for improving the interface friction coefficient was presented by adding Al 2219 alloy particles between the two Al sheets. Thus, the temperature of the weld interface could be improved at a comparatively low welding energy input. The reasons for the selection of Al 2219 particles as an interlayer are as follows. First, the hardness of Al 2219 particles (141.5 Hv) is higher than that of aluminum (34.6 Hv), which could activate the surfaces, and improve the friction coefficient in the faying interfaces. Second, Al 2219 particles could be easily combined during USW because of their relatively low melt point and good weldability. The effects of the interlayer thickness on the joint formation, microstructure, and mechanical strength of the Al/Al ultrasonic spot joints were systematically investigated.

## 2. Materials and Methods

**Figure 1:**
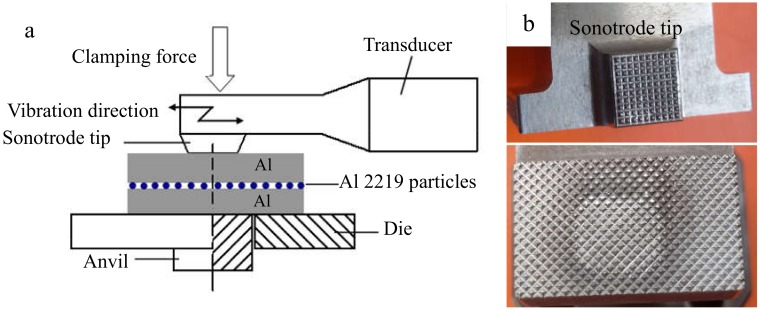
(**a**) Diagram of ultrasonic spot welding and (**b**) detailed knurl patterns of the sonotrode tip (top) and anvil (bottom).

**Figure 2:**
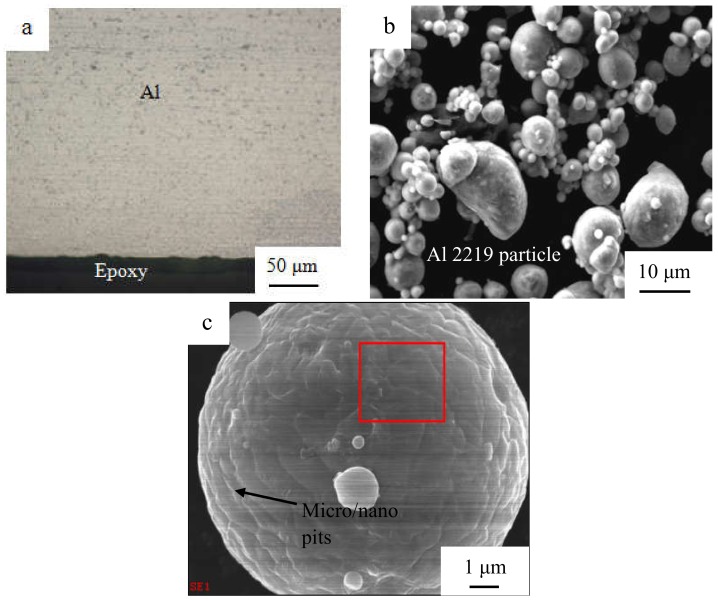
Optical microstructure (**a**) of the original 1100 aluminum sheet, a SEM micrograph of an Al 2219 particle (**b**), and a magnified SEM image of an Al 2219 particle (**c**).

**Figure 3:**
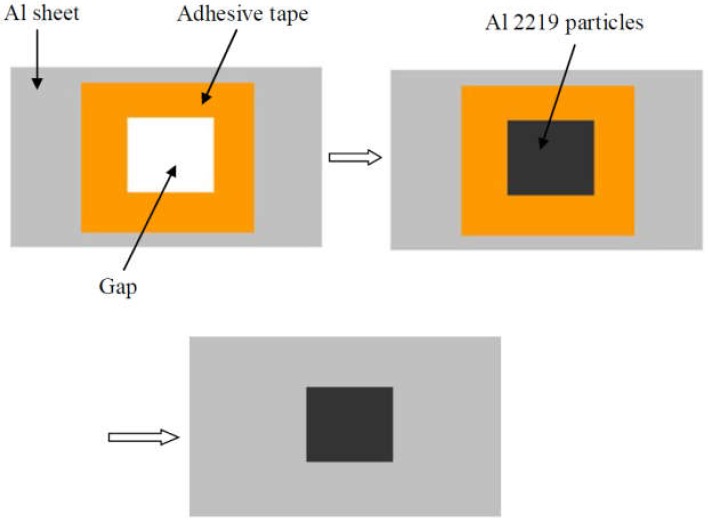
Processing schematic of the Al 2219 particle interlayer.

The base metal was an Al 1100 alloy sheet that had undergone hardening treatment with dimensions of 100 mm × 25 mm × 1.2 mm. Before the joining, the surfaces of the Al sheets were ground using 600# and 1000# sandpaper, and then, the specimens were rinsed with acetone. A SONICS MW-20 machine (Sonics, Newton, MA, USA) was employed for the welding, and the diagram was demonstrated in Figure 1a. The shape of the sonotrode tip was a square with 8-mm sides, and the detailed knurl patterns of the anvil and sonotrode tip are exhibited in Figure 1b. Al sheets were welded at the center of the 25-mm overlapped position. The vibration direction was perpendicular to the rolling direction of the sheets. The optical microstructure (OM) of the original 1100 aluminum sheet is shown in Figure 2a. A SEM micrograph of an Al 2219 particle is shown in Figure 2b, and the magnified image is shown in Figure 2c. The weight ratio of Al 2219 particles to alcohol in the mixture (Al 2219 particles and alcohol) was 1:4. First, a piece of tape was stuck onto the surface of the Al sheet, leaving a gap. Then, the mixture was put in the gap with a flat knife. Finally, the tape was torn off after the mixture was dry. The schematic is shown in Figure 3. Because the thickness of the tape was different (e.g., 10 µm, 20 µm), the specified interlayer thickness could be obtained. The average thickness of the Al 2219 particle interlayer was in the range of 10 to 80 μm at an interval of 10 μm. The composition of an Al 2219 particle is shown in Table 1.

**Figure 4:**
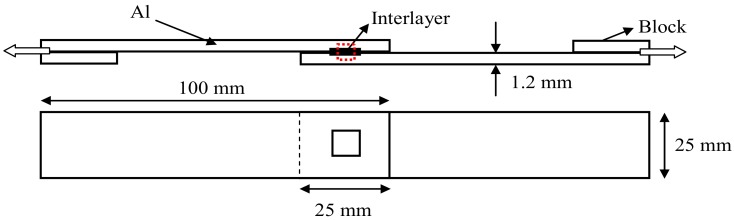
Diagram of welding sheet and design of the tensile shear test specimen.

The welding parameters employed for the fabrication were a welding time of 0.8 s, clamping pressure of 0.31 MPa, amplitude of 42 μm, and constant frequency of 20 kHz. A total of 5 joints were fabricated in each condition. One of them was used for microstructure examination, and the others were employed for lap shear tensile tests. In fact, the welding time was optimized in the experiment. It was found that the optimal welding time (0.6–0.8 s) was beneficial to the weld interface bonding, whereas beyond the critical value (0.8 s), the aluminum beneath the sonotrode tip would be squeezed out, and the sonotrode sticking phenomenon was present. In this paper, the optimal welding time (0.8 s) was chosen as a constant. The microstructure was examined employing an optical microscope (OM, GX71, OLYMPUS, Tokyo, Japan), scanning electron microscope (SEM, FEI, Hillsboro, OR, USA) equipped with energy dispersive X-ray spectroscopy (EDS). To assess the joint strength and identify the optimal condition, lap shear tensile strength was achieved by employing a tensile tester at a constant strain rate of 1 mm/min in room temperature conditions. During the tensile tests process, blocks were used to reduce the joints rotation and sustain the shear loading as long as possible, as demonstrated in Figure 4. The weld temperatures were measured using 0.5-mm diameter k-type thermocouples placed just below the top sheet surface, using a precision drilled hole to connect the bottom sheet’s top surface (weld interface position).

## 3. Results and Discussion

From Figure 2a, it can be seen that no asperities or grooves occur near the surface regions, and the elongated 1100 aluminum grain is parallel to the rolling direction. In Figure 2b,c, it can be observed that the average diameter of the spherical Al 2219 particle (141.5 Hv) was 8 μm. Some micro/nano pits appear on the surface of the Al 2219 particle, resulting in the improvement of the friction coefficient at the weld interface.

**Figure 5:**
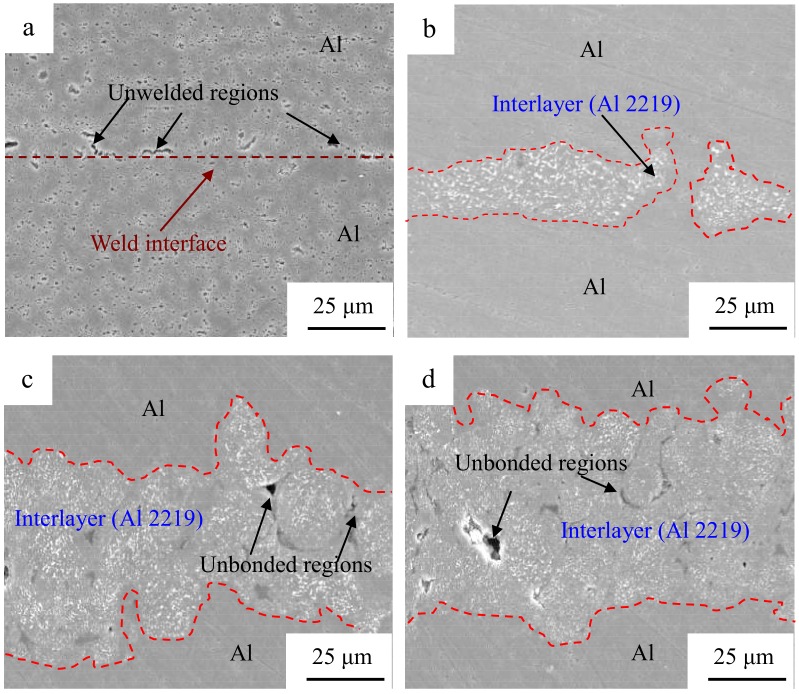

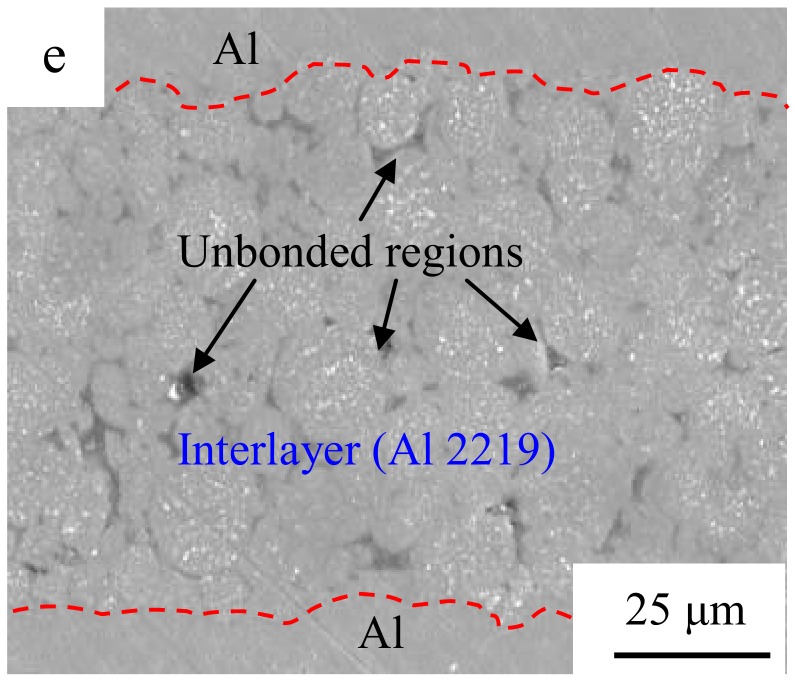
SEM microstructure of the cross section of the Al/Al joints with different interlayer thicknesses: (**a**) without an interlayer; (**b**) 10 μm; (**c**) 30 μm; (**d**) 60 μm, and (**e**) 80 μm.

**Figure 6:**
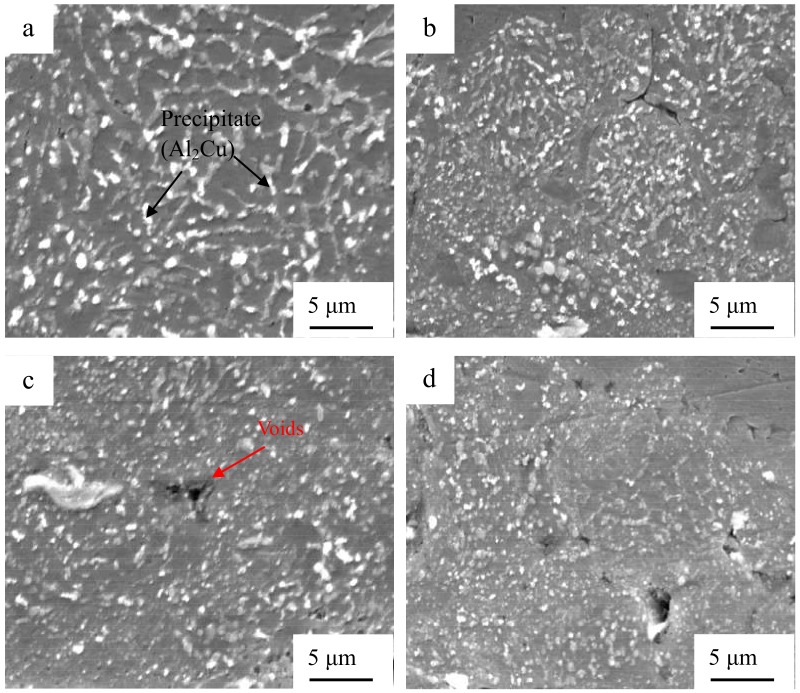
Precipitates from Al 2219 for the joints with different interlayer thicknesses: (**a**) 10 μm; (**b**) 30 μm; (**c**) 60 μm, and (**d**) 80 μm.

Figure 5 indicates the SEM microstructure of the cross section of the Al/Al joints with different interlayer thicknesses. The specimen fabricated without an interlayer (Figure 5a) possessed some gaps in the weld interface where bonding was not obtained. When the interlayer thickness reached 10 μm (Figure 5b) and the weld interface was clean, waved, and free of gaps, strong interfacial bonding was obtained. By further increasing the interlayer thickness, the unbonded regions and voids increased in the Al 2219 particle interlayer, as demonstrated in Figure 5c–e. The size and amount of the precipitate (Al_2_Cu) decreased in the interlayer, as shown in Figure 6. Thus, considering the abovementioned, the interlayer thickness is important to the microstructure of the joints.

**Figure 7:**
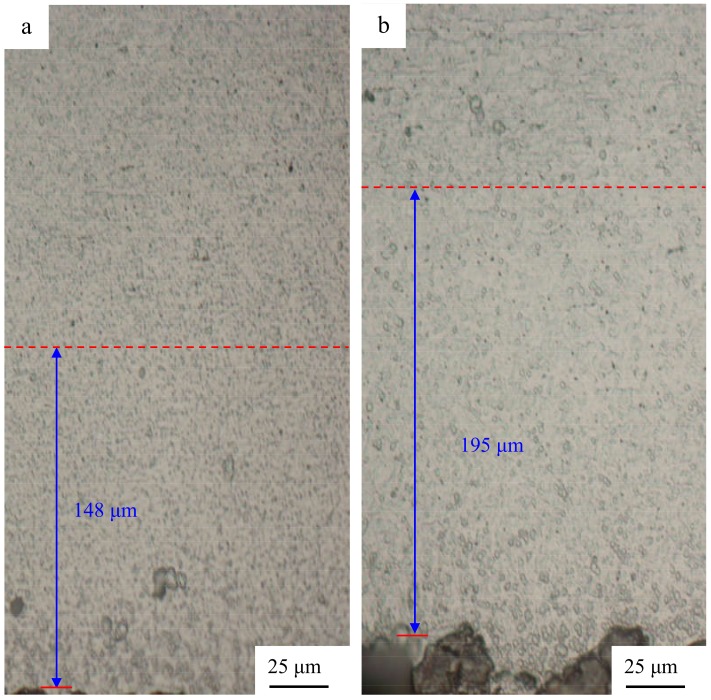

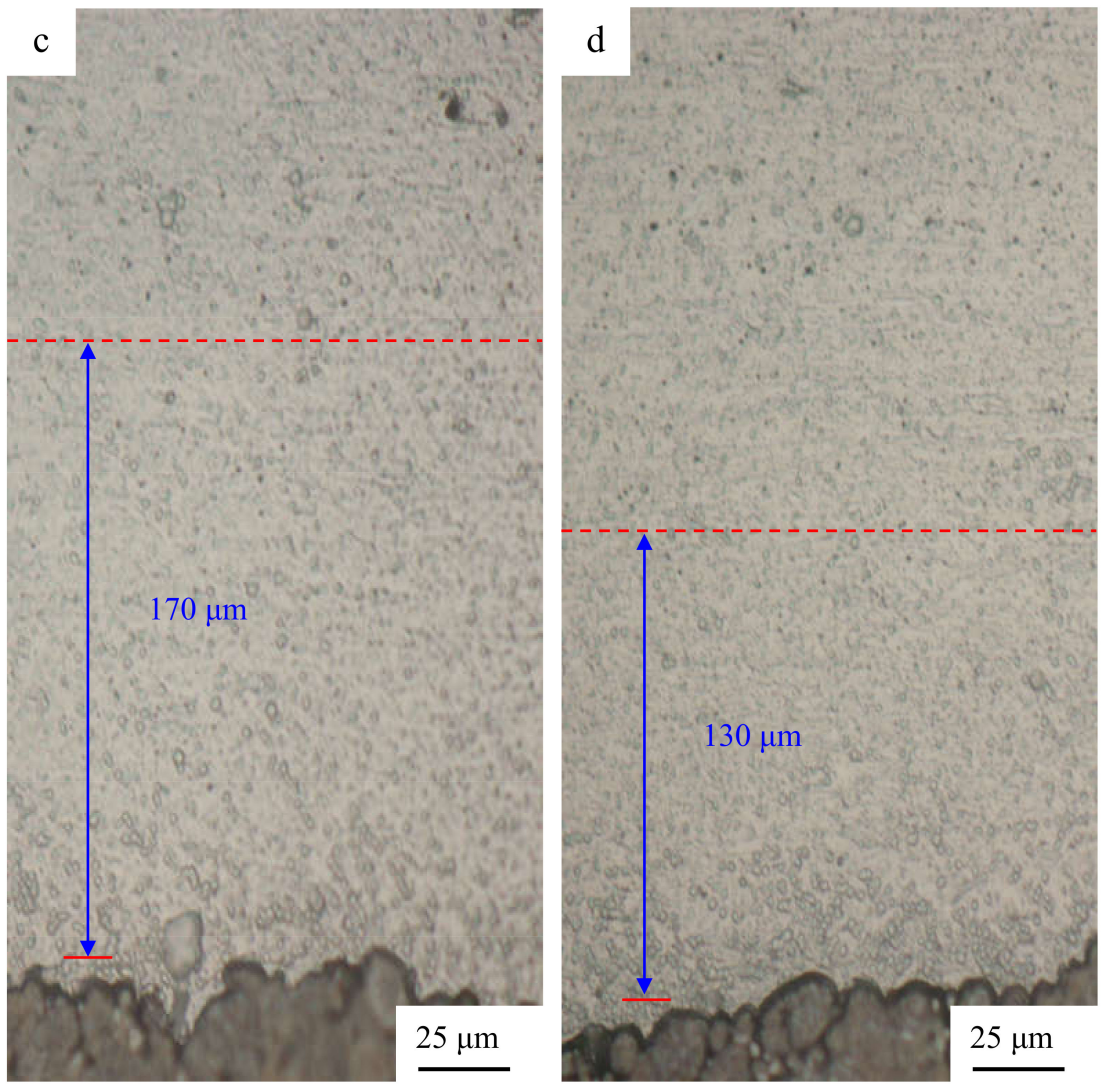
Optical microscope (OM) images of the Al side for Al/Al joints with interlayers of different thicknesses: (**a**) without an interlayer; (**b**) 10 μm; (**c**) 30 μm, and (**d**) 60 μm.

To obtain a deeper understanding of the microstructure evolution of the sheets during USW, OM was carried out. The characteristics of the microstructure around the weld interface can identify the phenomena taking place during the weld formation. The original elongated grains (in Figure 2a) disappear, and the recrystallized microstructure and grain growth occur along the weld interface, as seen in Figure 7a–d. When the interlayer thickness was 10 μm, the thicknesses of the decreased elongated aluminum grains (the section between the red lines), as shown in Figure 7b (195 μm), were thicker than those shown in Figure 7a (148 μm), 7e (170 μm), and 7f (130 μm). It is confirmed that aluminum grain growth takes place at the weld interface, and the Al 2219 particle interlayer is beneficial to grain growth. 

**Figure 8:**
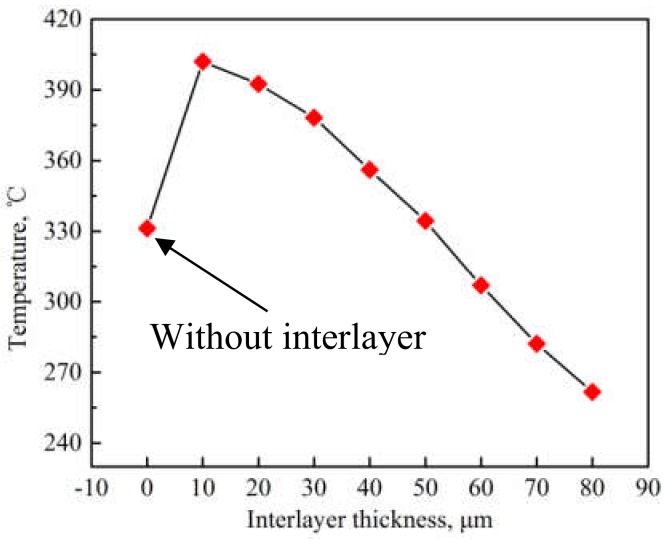
Temperature of the weld interface for the joints with different interlayer thicknesses.

**Figure 9:**
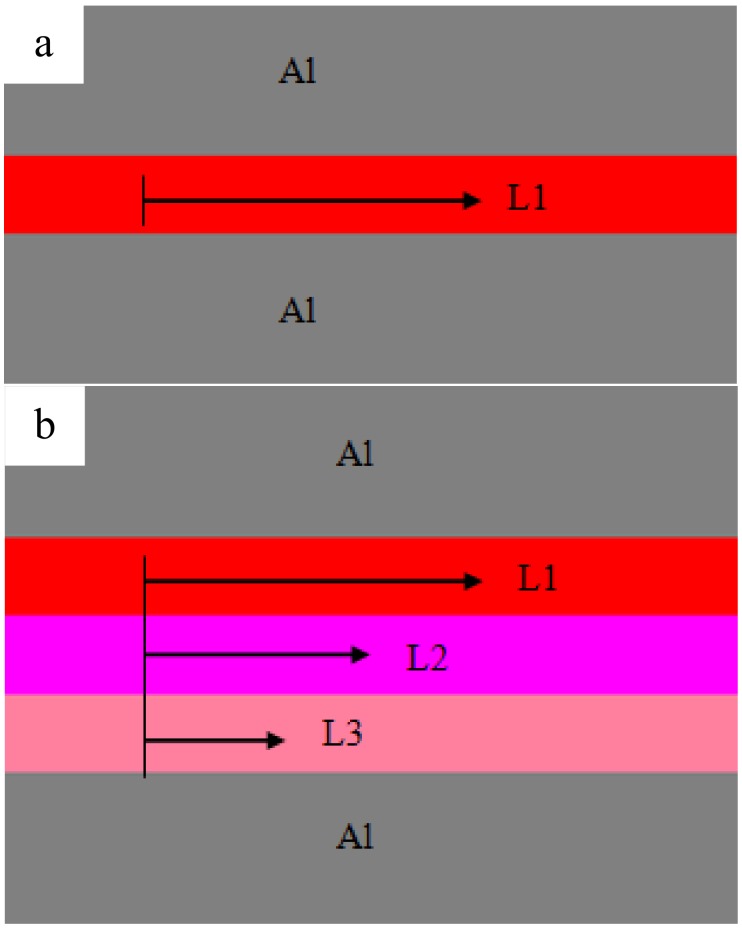
Schematic of the vibration amplitude of the Al 2219 particles in the joints with different interlayer thicknesses: (**a**) 10 μm and (**b**) 30 μm. (The 10-μm thickness interlayer is equivalently composed of a layer of Al 2219 particles, and the 30-μm interlayer thickness equivalently consists of three layers of Al 2219 particles.).

In order to identify the reasons for the microstructure evolution in the weld interface, thermal measurements were achieved. Figure 8 exhibits the temperature of the weld interface for the joints with different interlayer thickness. It can be observed that when the interlayer thickness is 10 μm, the temperature of the weld interface reaches the maximum (402 °C), which is lower than the melting point of the aluminum alloy. By further increasing the interlayer thickness, the weld interface temperature decreases. It can be concluded that an appropriate interlayer thickness is beneficial to enhance the heat generation and the weld interface temperature. The reasons can be summarized as follows. Frictional behavior between the two metal sheets is critical to the formation of pure metallurgical bonding in the weld interface [25,26]. Transverse movements of each layer of Al 2219 particles during USW is different, and the vibration amplitude of the top layer of Al 2219 particles precisely increases under the action of the sonotrode tip movement. Then, the vibration of the other layers take placed sequentially, and the vibration amplitude of the rest of the layers of Al 2219 particles gradually decreases due to the energy loss during the welding process. The schematic of this process is shown in Figure 9. A large vibration amplitude accelerates the heat generation at the weld interface. By increasing the thickness of the Al 2219 particle interlayer, the heat generation in the bottom layer of the Al 2219 particle interlayer decreases. Consequently, the weld interface temperature decreases as the thickness of the Al 2219 particle interlayer increases, as demonstrated in Figure 8. The higher temperature is beneficial to the free flow of the material and precipitation from interlayer. Therefore, the occurrences of the phenomenon in Figure 5, Figure 6 and Figure 7 can be demonstrated. The grain growth is attributed to the increase of the weld interface temperature, as well as the severe cold working.

During USW, heat generation, resulting in the increase of the temperature at the weld interface, is obtained from plastic deformation heating, frictional heating at the weld interface, and possible acoustic heating from the ultrasonic wave, as demonstrated by Panteli et al. [27]. The generated friction heat is due to the large vibration amplitude between the faying interfaces of the two Al sheets. The vibration frequency is 20 kHz, and the increasing amplitude leads to the increase of velocity, which can have an active role in the decrease of the yield strength of the Al sheet. Under the combination effects of the clamping force and shear vibration, the local area of Al sheets becomes soft, leading to the generation of severe plastic deformation. As the welding process progresses, the yield strength of the Al sheet decreases because of the plastic deformation heat. In addition, the Al sheets bear cyclic deformation at 20 kHz. It takes place at a strain rate of 1000 when the welding time is less than 1 s, and the material is subjected to 20,000 deformation cycles at a high strain rate of 1000. The number of microstructure defects (vacancy and dislocation) are presented at the weld interface, because of the high strain rate dynamic deformation and severe plastic deformation [21]. An ultrasonic wave can be effectively spread in the complete metal lattice, but it is inclined to absorption in the microstructure defects [28,29,30]. The primarily absorbed acoustic energy in the microstructure defects promotes the softening of the Al sheet and reduces the distinct welding stress of the joints. Dislocations are activated from the absorption of the acoustic heat and glided with the improved mobility of the atoms; thus, the flow of the material becomes easier, leading to the generation of severe plastic deformation. Carboni et al. [31] demonstrated that plastic deformation can further contribute to the enhancement of the weld interface temperature because of the consumption of plastic deformation heat. Because of the appropriate addition of the Al 2219 particle interlayer (10 μm, 20 μm, and 30 μm), the friction coefficient of the contacted surfaces for the two Al sheets can be improved, leading to the generation of friction heat, and then, the heat generation attributed to the absorbed ultrasonic energy and plastic deformation is enhanced. Therefore, when the interlayer thickness is 10 μm, 20 μm, or 30 μm, the weld interface temperature is higher than that of the other joints. 

**Figure 10:**
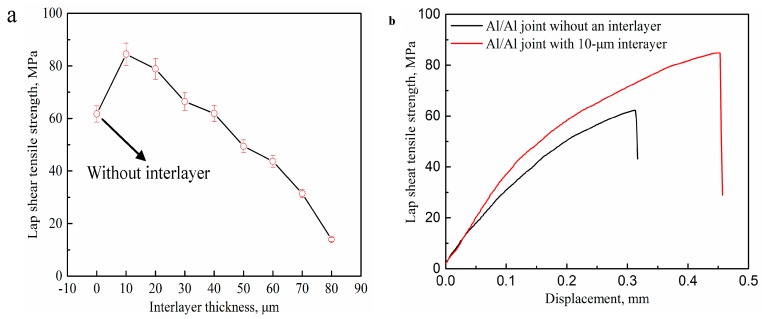
(**a**) Relationship between the lap shear tensile strength and the interlayer thickness for the ultrasonic spot-welded (USWed) Al/Al joints; (**b**) the load-displacement curves for the joints without an interlayer and with a 10-μm interlayer.

Figure 10 demonstrates the relationship between the lap shear tensile strength and the interlayer thickness for the USWed Al/Al joints and the load-displacement curves for the joints without an interlayer and with a 10-μm interlayer. It can be observed that the lap shear tensile strength first increases up to the maximum and then has a decreasing tendency with the further increasing of the interlayer thickness. When the interlayer thickness is 10 μm, the maximum lap shear tensile strength is 84.8 MPa, which is 36 % higher than that of the joint without an interlayer. It is confirmed that the interlayer thickness is critical to the lap shear tensile strength of the Al/Al 2219/Al joints.

As soon as the shear vibration and the clamping force are exerted during USW, the mutual rubbing and plastic deformation on the faying surfaces of the two Al sheets will occur. Al 2219 particles with a hardness of 141.5 Hv are conductive to activate the Al sheet surfaces to generate fresh metal contacts at the weld interface. Atomic forces can play an important part when the contact interfaces are close enough together. Prior to the welding process, there are numerous micro-asperities on the surfaces of the Al sheet and Al 2219 particles, and the first mutual contact takes place on the micro-asperity surfaces, resulting in un-contacted regions in the Al/Al 2219 interface. The combination in the un-contacted regions cannot take effect until these voids are eliminated and the surfaces contact strongly. This is where the interfacial plastic deformation plays a crucial role, accelerating the materials to extrude into the un-contacted areas. As the welding progresses, the size and number of the bonded areas increase, resulting in more effectively net contacted areas. As can be seen in the results shown in Figure 8, the highest weld interface temperature is obtained when the thickness of the interlayer is 10 μm, resulting in the softening, severe plastic deformation and sound weldability of the Al sheet. Therefore, the interface of Al/Al 2219 is complexly intertwined together (Figure 5b), which promotes the improvement of the tensile strength of the Al/Al 2219/Al joint. The mechanical strength of the USWed joints is a comprehensive effect of mechanical interlocking, micro-joints, and interfacial waves along the weld interface. It is concluded that the weld interface is sound. If an interlayer in the thickness range of 20–80 μm or no interlayer is placed between the two Al sheets, the weld interface temperature is lower than that of a joint with a 10-μm interlayer (Figure 8), leading to the generation of unbonded areas in the interlayer (Figure 5e), and then, joining is prevented. Poorly combined regions in the interlayer or weld interface are the crack sources, which take place in the beginning phase of the load transition process and then promote the crack growth until an eventual fracture occurs. Thus, the lap shear tensile strength of the joint with the 10-μm interlayer is better than that of the other joints.

**Figure 11:**
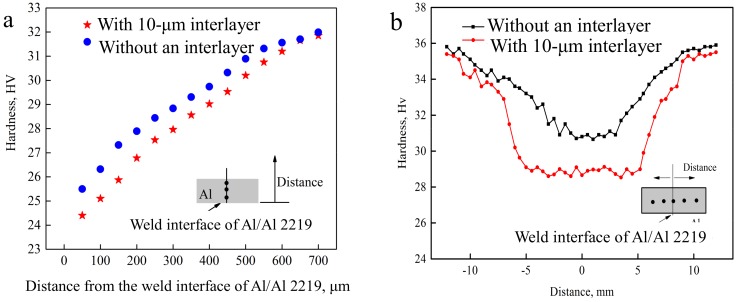
Vickers characteristic microhardness profiles of the Al side in the USWed joints with different thicknesses of interlayers; (**a**) vertical in the weld center; (**b**) horizontal.

Figure 11 shows the Vickers characteristic microhardness profiles of the Al side in the USWed joints with different thicknesses of interlayer (a) cross sections along the thickness direction of the aluminum sheet and (b) cross sections in the middle of the aluminum sheet. It is seen from Figure 11a that by increasing the distance from the weld interface of Al/Al 2219 along the thickness direction of the Al sheet, the hardness increases until the distance is 700 μm, demonstrating that the peak temperature is at the center of the nugget. Thus, recrystallization and grain growth occur. In Figure 11b, lower hardness areas are located in the center of the weld regions for the joints with different thicknesses of interlayers. The hardness for the joint with the 10-μm interlayer is lower than that of the joint without an interlayer. This is attributed to plastic deformation heating, frictional heating at the weld interface, and possible acoustic heating from the ultrasonic wave taking place at the weld interface. Then, the temperature is enhanced at the weld interface, facilitating the recrystallization and grain growth. Thus, the microhardness of the Al sheet decreases depending on the Hall–Petch-type relationship as reported by Hall. Meanwhile, the more complete the recrystallization process and grain growth, the softer the sheets [19]. 

**Figure 12:**
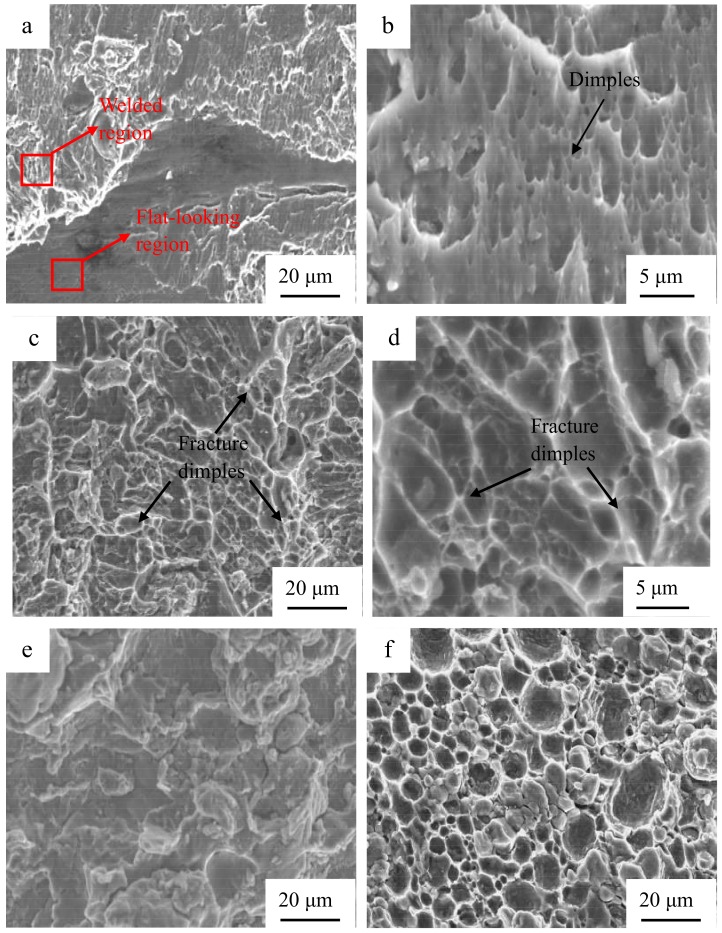

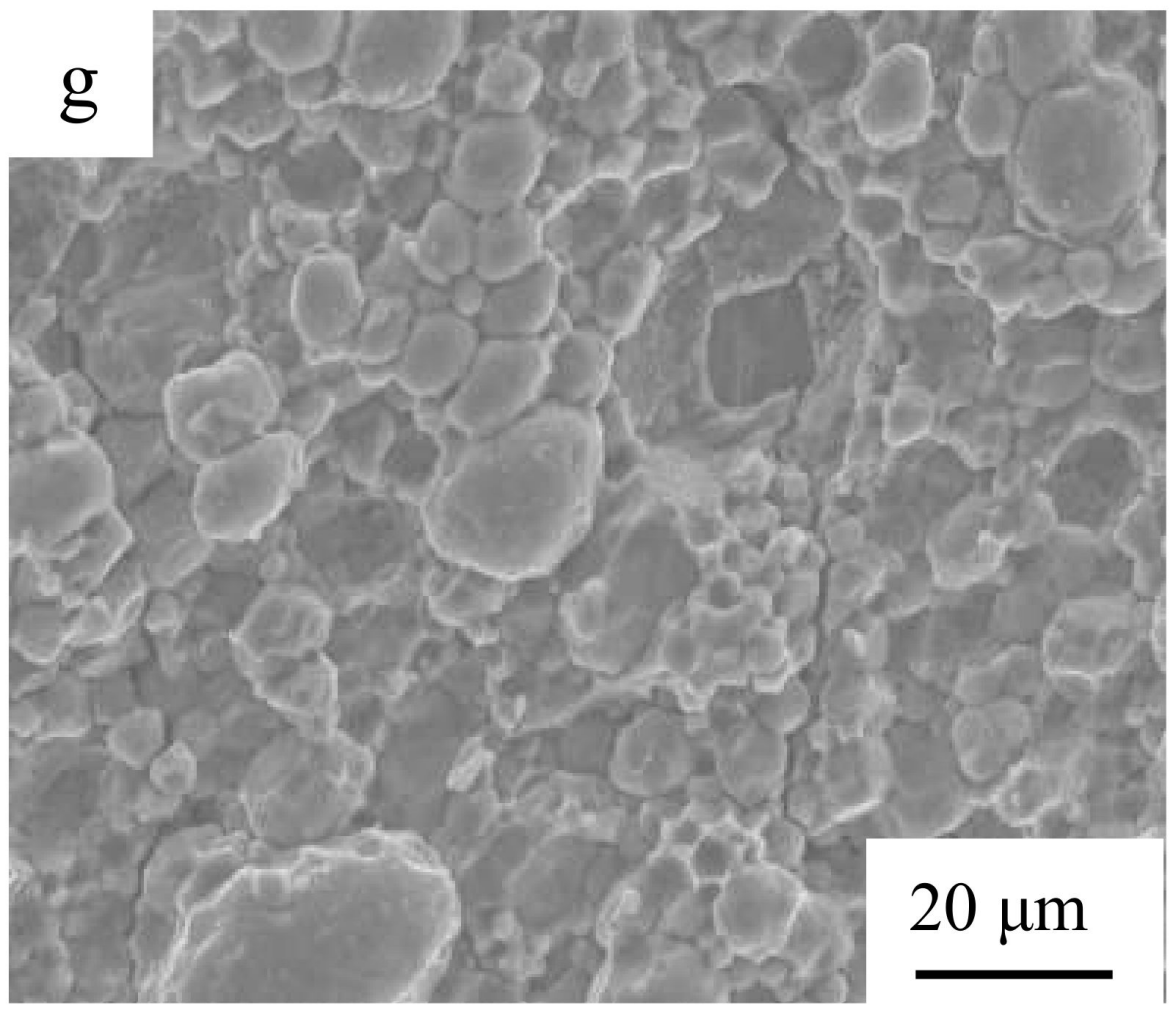
Fracture morphology of the Al/Al joints with different interlayer thicknesses, (**a**) without an interlayer, (**c**) 10 μm, (**b**,**d**) showing the magnified areas in (**a**,**c**), respectively, (**e**) 30 μm, (**f**) 60 μm, and (**g**) 80 μm.

**Figure 13:**
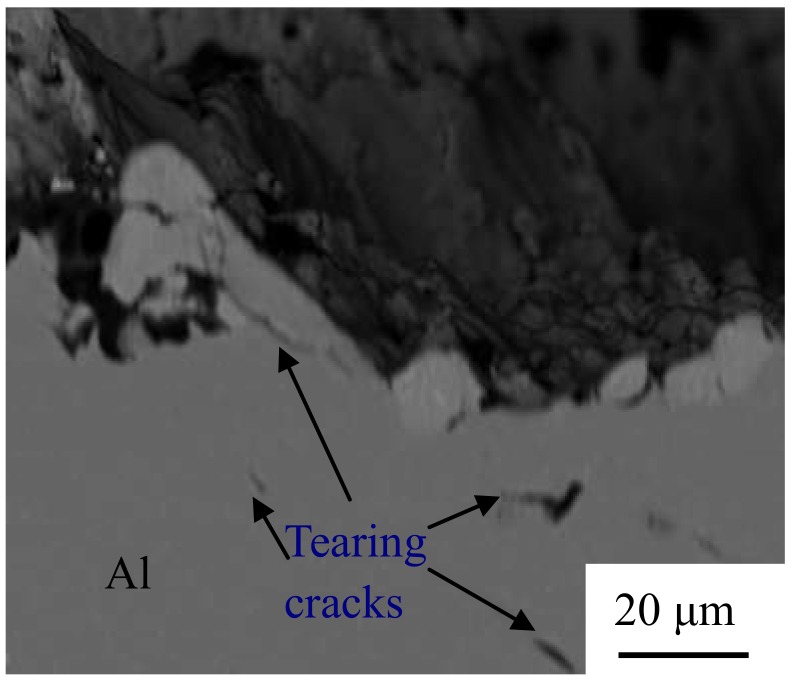
Flank face of the fracture path for the Al/Al joint with the 10-μm interlayer.

Figure 12 indicates the fracture morphology of the Al/Al joints with different interlayer thicknesses. Figure 12b,d shows the magnified SEM images corresponding to Figure 12a,c, respectively. Some localized flat-looking type areas were exhibited in the fracture surface (Figure 12a), demonstrating that some unwelded areas existed at the weld interface. The size of the fracture dimples was around 1 μm (marked by the arrow in Figure 12b), which is indicative of a ductile fracture. Few unwelded areas were found, as seen in Figure 12c, and some fracture dimples with a size of around 5 μm were present in the fracture surface, as seen in Figure 12d, confirming that it is a highly ductile fracture and the interfacial bonding of Al/Al 2219 is sound. As the interlayer thickness increases, the dimples disappear gradually, as shown in Figure 12e–g. More fracture dimples (Figure 12c,d) take place in the fracture surface of the joint with the 10-μm interlayer, indicating that the fracture occurs through the void formation/nucleation, propagation, and coalescence. Figure 13 indicates the flank face of the fracture path for the Al/Al joint with the 10-μm interlayer. There are some tearing cracks and dimples at the Al side, demonstrating that large tensile deformation takes place in the joint. It is in accordance with the fracture morphology in Figure 12c,d. In addition, it is also demonstrated that the bonding interface of Al/Al 2219 for the joint with the 10-μm interlayer is sound, which is consistent with the results demonstrated in Figure 10.

## 4. Conclusions

Al/Al joints with interlayers of different thicknesses were fabricated employing ultrasonic spot welding. The conclusions are summarized below:

1. The optimal thickness of the Al 2219 particle interlayer is 10 μm, which is beneficial to enhance the weld interface temperature up to 402 °C.

2. The peak lap shear tensile strength (~84.8 MPa) for the joint with the 10-μm interlayer is 36% higher than that (~67.3 MPa) for the joint without an interlayer.

3. For the joint with the 10-μm Al 2219 particle interlayer, the bonding interface of Al/Al 2219 with a wave-like shape is good, and no significant diffusion layer occurs.

4. The strengthening mechanism is caused by the increase of the plastic deformation and the contacted areas at the weld interface.

## Figures and Tables

**Table 1:** Chemical composition of an AL 2219 particle.

Cu	Mn	Si	Fe	Mg	Zn	Ti	Al
5.8–6.8	0.2–0.4	0.20	0.20	0.02	0.10	0.02–0.10	Bal
